# Nuclear proteins and diabetic retinopathy: a review

**DOI:** 10.1186/s12938-024-01258-4

**Published:** 2024-06-25

**Authors:** Bin Li, Wahab Hussain, Zhi-Liang Jiang, Jia-Yi Wang, Sarfraz Hussain, Talat Bilal Yasoob, Yuan-Kun Zhai, Xin-Ying Ji, Ya-Long Dang

**Affiliations:** 1 Department of Ophthalmology, The First Affiliated Hospital, Henan University, Kaifeng, 475004 Henan China; 2 School of Stomatology, Henan University, Kaifeng, 475000 China; 3 Kaifeng Municipal Key Laboratory for Infection and Biosafety, Henan International Joint Laboratory of Nuclear Protein Regulation, School of Basic Medicine Science, Henan University, Kaifeng, 475000 China; 4 School of Clinical Medicine, Henan University, Kaifeng, 475004 Henan China; 5 San-Quan College, XinXiang Medical University, No. 688 Xiangyang Road, Hongmen Town, Hongqi District, Xinxiang City, Henan, 453003 China; 6 College of Environment, Hohai University, Nanjing, 210098 China; 7 Department of Animal Sciences, Ghazi University, Dera Ghazi Khan, 32200 Pakistan; 8 Kaifeng Key Laboratory of Periodontal Tissue Engineering, Kaifeng, 475000 China; 9 Faculty of Basic Medical Subjects, Shu-Qing Medical College of Zhengzhou, Mazhai, Erqi District, Zhengzhou, 450064 Henan China; 10 Department of Ophthalmology, Sanmenxia Central Hospital, Henan University of Science and Technology, Sanmenxia, Henan China; 11 Department of Ophthalmology, Sanmenxia Eye Hospital, Sanmenxia, Henan China; 12 Department of Ophthalmology, Henan University of Science and Technology School of Medicine, Luoyang, Henan China

**Keywords:** Diabetic retinopathy, Nuclear protein, Non-proliferative diabetic retinopathy

## Abstract

Diabetic retinopathy (DR) is an eye disease that causes blindness and vision loss in diabetic. Risk factors for DR include high blood glucose levels and some environmental factors. The pathogenesis is based on inflammation caused by interferon and other nuclear proteins. This review article provides an overview of DR and discusses the role of nuclear proteins in the pathogenesis of the disease. Some core proteins such as MAPK, transcription co-factors, transcription co-activators, and others are part of this review. In addition, some current advanced treatment resulting from the role of nuclear proteins will be analyzes, including epigenetic modifications, the use of methylation, acetylation, and histone modifications. Stem cell technology and the use of nanobiotechnology are proposed as promising approaches for a more effective treatment of DR.

## Introduction

### Diabetic retinopathy (DR)

Diabetic retinopathy (DR) is a common microvascular complication of diabetes mellitus and the main cause of visual loss in older people. In the early stages of DR, hyperglycemia and altered metabolic pathways lead to oxidative stress and the development of neurodegeneration [1]. Vascular endothelial damage, the development of microaneurysms, and punctate intraretinal hemorrhage are early hallmarks of non-proliferative diabetic retinopathy (NPDR).The disruption of the blood–retinal barrier and release of several inflammatory cytokines and plasma proteins lead to the hard exudates observed on fundoscopy [2]. As the disease progresses, vasoconstriction and capillary occlusions lead to crushed capillaries and ischemia of the retina. The presence of ‘absorbent cotton spots’ can be recognized in this stage. In the final stage of diabetic retinopathy, severe hypoxia leads to neovascularization (NV), vitreous hemorrhage, and retinal detachment [3].

Diabetes can have a significant impact on the eyes and lead to various changes that distinguish a diabetic eye from a healthy eye. These changes often lead to diseases, such as diabetic retinopathy, diabetic macular edema, and glaucoma [4]. Retinal changes one of the most common complications of diabetes is diabetic retinopathy, which leads to damage to the blood vessels in the retina [5]. A study published in Diabetes Care found that people with diabetic retinopathy have characteristic retinal changes, including microaneurysms, hemorrhages, and exudates, compared to healthy eyes [6]. In another study, increased macular thickness and changes in the retinal layers were cited as the main features of diabetic macular edema, highlighting the structural changes in the eye caused by diabetes [7].

Diabetic retinopathy is an eye disease that leads to blindness and loss of vision in diabetics [8]. In industrialized countries, diabetic retinopathy is actually the main cause of blindness. Studies suggest that around 80% of people with type 1 or type II diabetes suffer from DR [9, 10]. In DR, the high blood sugar levels associated with diabetes damage the blood vessels in the retina. This disruption of normal blood flow in the retina can have serious effects on vision. DR can be divided into two types. Non-proliferative diabetic retinopathy (NPDR) is usually associated with the early stages of DR. At this stage, restrictions in blood flow, whether due to vascular changes or blockage, can result in the retina not being adequately supplied with nutrients and oxygen. In addition, tiny blood vessels can swell and penetrate the retina. These processes lead to damage to the retinal tissue and swelling of macular (macular oedema), which ultimately leads to loss of vision [11]. As the disease progresses, it becomes proliferative (PDR). At this point, the blood vessels are damaged to such an extent that they become blocked, leading to the growth of new, abnormal, blood vessels in the retina (neovascularization). These unstable new blood vessels have several serious effects on vision: they can bleed and allow fluid to enter the eye; they lead to the formation of scar tissue, which can cause the retina to detach; and they can lead to an increase in pressure in the eye, which can damage the optic nerve [8, 12].

### Risk factors for diabetic retinopathy

The main risk factors for DR are poor blood sugar control, the duration of diabetes, and high blood pressure [13]. For example, 25% of people with type 1 diabetes have some degree of DR after five years, and 2% are diagnosed with PDR within this period. After 15 years of diabetes, 80% have some degree of DR and 25% have PDR. Other risk factors for DR include abnormal blood lipids, kidney disease, smoking, and a high body mass index [14]. Some studies have also reported that a combination of genetic and environmental factors is responsible for the development of DR in humans [15]. In terms of genetic risk factors, the variants associated with an increased risk of type 2 diabetes are also associated with DR [16]. Genetic factors associated with the development of diabetic retinopathy mutations or variations in genes involved in glucose and lipid metabolism, such as the gene for insulin (INS) or the transcription factor 7-like 2 (TCF7L2), have been associated with an increased risk of diabetic retinopathy [17]. The RAAS is a hormonal system that regulates blood pressure and fluid balance. Genetic variations in genes such as angiotensinogen (AGT), angiotensin-converting enzyme (ACE), and angiotensin II receptor type 1 (AGTR1) have been associated with an increased risk of diabetic retinopathy [18]. Several genes involved in inflammatory processes and oxidative stress are associated with diabetic retinopathy. These genes include vascular endothelial growth factor (VEGF), interleukin-6 (IL-6), and superoxide dismutase (SOD) [19]. Numerous variations in the VEGFC gene (vascular endothelial growth factor C) are associated with an increased risk of progressive macular oedema [20]. Other genetic risk factors include people with Downsyndrome. Regular eye infections and severe eye infections in early childhood have also been associated with an increased risk of DR [21]. Non-genetic factors can significantly influence the development and progression of diabetic retinopathy, a complication of diabetes that can lead to vision loss. These factors may include blood glucose control, as poor control of blood sugar levels onset to the occurrence and severity of retinopathy [22]. High blood pressure has been identified as a risk factor for diabetic retinopathy, and controlling blood pressure through medications, lifestyle changes, and regular monitoring can help reduce the risk and delay the progression of retinopathy [23]. Elevated cholesterol and triglyceride levels can contribute to the development and worsening of retinopathy [24]. Smoking and excessive alcohol consumption are associated with an increased risk and progression of diabetic retinopathy [25, 26].

### Prevalence of diabetic retinopathy

In 1992, Klein [27] presented the results of the Wisconsin Epidemiological Study of Diabetic Retinopathy (WESDR study), a population-based study conducted in southern Wisconsin involving 1370 patients diagnosed with diabetes and 996 insulin-dependent younger diabetics (diabetes diagnosed under 30 years of age). The study used standard protocols to determine the prevalence and severity of diabetic retinopathy and the associated risk factors. PDR was found in 23% of the younger-onset group, 10% of the older-onset group receiving insulin, and 3% of the group not receiving insulin. In 1995, Klein investigated [28] reported that after a 10-year period, the incidence of macular oedema was 20.1% in the younger-onset group, 25.4% in the older-onset group receiving insulin, and 13.9% in the older-onset group not receiving insulin. In 1998, the United Kingdom Prospective Diabetes Study (UKPDS) analyzed the baseline level of retinopathy in 2964 newly diagnosed patients with type 2 diabetes. Retinopathy, defined as microaneurysms or worse lesions in at least one eye, was found in 39% of men and 35% of women, and visible retinopathy with absorbent cotton spots or intraretinal microvascular abnormalities was present in 8% of males and 4% of women [29]. In 2002, Younis [30] reported the baseline results of a population screening in Liverpool, UK, involving 831 people with type 1 diabetes and 7231 people with type 2 diabetes. The results showed that of the type 1 diabetes, 45.7% had DR, 3.7% had PDR, and 16.4% had eye disease serious enough to threaten their sight. Individual case studies suggest that children can develop pre-proliferative DR or diabetic retinopathy as early as 12 years of age, with a diabetes duration of about 5.6 years [31]. Numerous studies on diabetic eye disease have been conducted in different regions of the world, contributing to a picture of growing concern about the prevalence of this condition [32, 33]. In addition, screening for type 2 diabetes showed a significantly lower proportion of DR (7.6% and 6.8%, respectively) in a smaller proportion of people screened positive for type 2 diabetes than in the known diabetes population than in the known diabetes population [34, 35]. According to the World Health Organization, there are more than 37 million cases of blindness in the worldwide [36]. The prevalence of DR in Pakistan was reported to be 28%, with 9% of patients having eye disease severe enough to threaten their sight. The prevalence of DR in Pakistan was reported to be 28%, with 9% of patients having eye disease severe enough to threaten their sight [21, 38]. The same study also reported that the prevalence of DR ranges from 10.6% to 91.34% [38], and that sight-threatening diseases very between 4% and 46% [20, 23]. The prevalence of DR in diabetic worldwide is 27.0% [39], but region-specific studies indicate a prevalence of 31.6% in Africa and 19.48% in Ethiopia [40] (Table 1). There are 95 million diabetics worldwide who suffer from DR. One third of them are thought to have DR so severe that it threatens their vision [41]. The annual incidence of DR is between 2.2% and 12.7%, and progression is between 3.4% and 12.3% [18, 26]. Nevertheless, the global prevalence of blindness was reported 1.5 million and the prevalence of DR at 0.4 million [42]. This prevalence was reduced bynutritional, social, and medical support, and these factors also slow the progression of DR. This suggests that working on environmental factors may help to reduce the prevalence of DR in diabetics [43].

**Table 1:** Prevalence of DR in a diabetic patient within different countries

Countries	Prevalence of DR %	Reference
United Kingdom	28	(González, Johansson, Wallander, & Rodríguez 2009)
Russia	*1.9*	(Bikbov et al. 2019)
Switzerland	*4.9*	(Glass et al. 2006)
Australia	22.2	(Landers, Henderson, & Craig 2010)
Pakistan	56.9	(Sohail 2014)
Japan	18.9	(Fukushima et al. 2022)
China	27.9	(Zhang, Chen, Chen, & Zhang 2017)

### Symptoms and pathogenesis of diabetic retinopathy

Symptoms of DR include blurred vision, spots or dark streaks in the vision, loss of vision [44], dark or blank areas, sometimes blurred vision, a series of floaters in the field of vision, blurred vision, and others. In addition, some patients show only one symptom, while others show a combination of two or more symptoms [45]. As people with DR often show no symptoms at the beginning of the disease, regular eye examinations are crucial for early detection and rapid action. This is of course particularly important for people who are known to have diabetes.

**Fig. 1:**
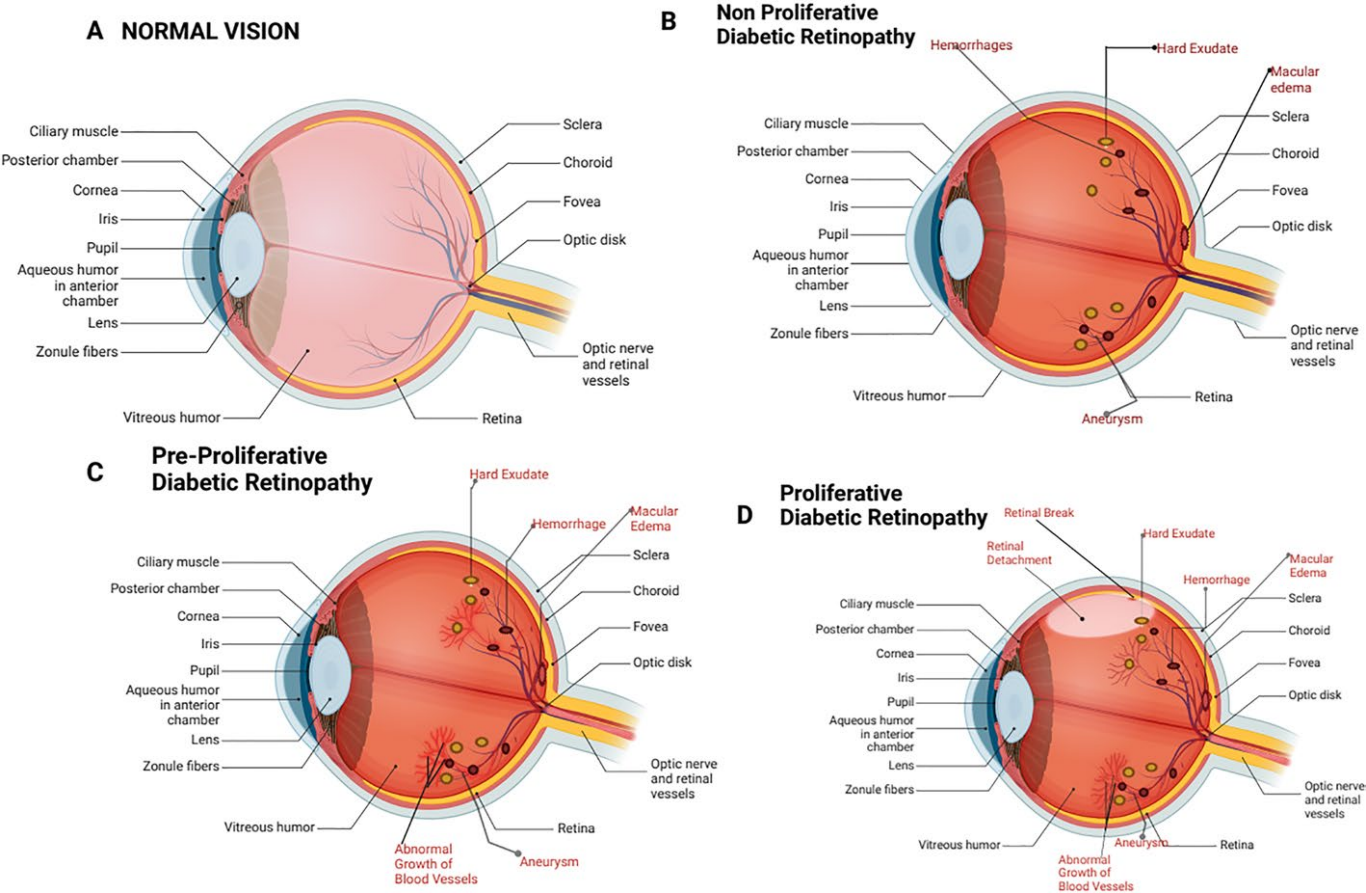
Diabetic retinopathy's clinical pathogenesis. **A.** Normal vision: lacks any signs of visual impairment. **B.** non-proliferative diabetic retina: characterized by tissue hypoxia, vascular leakage, thickening of the basement membrane, loss of pericyte, and variations in blood flow. **C. **Pre-proliferative diabetic retina: soft exudates, oedema, and hypoxia. **D.** Proliferative diabetic retina: blindness, retinal detachment, fibrovascular ridges

The clinical pathogenesis is based on the presence of visible ophthalmoscopic retinal microvascular lesions in individuals suffering from type 1 or type 2 diabetes. The disease then progresses over time through three stages (non-proliferative, pre-proliferative, and proliferative DR), as shown in Fig. 1. Other problems that can develop in patients with DR generally include: cascades, glaucoma, macular oedema, and retinal detachment. Glaucoma is a result of increased pressure in the eye caused by the growth of new blood vessels, while macular oedema is caused by fluid from damaged blood vessels entering the retinal area. Retinal detachment can occur when scarring of the newly grown blood vessels causes part of the retina to detach from the back of the eye socket.

A number of different metabolic pathways are involved in the development of vascular damage caused by hyperglycemia [46], and these include advanced gyrations, the polio pathway, the hexamine pathway, protein kinase, and others. Changes that occur in the earliest stages due to hyperglycemia include dilation of blood vessels and changes in blood flow [47]. The loss of pericytes and the formation of microaneurysms are also early changes in the retinas of diabetics [48]. As the pericytes form the structure to the capillaries, their loss leads to an expansion of the capillary walls. The pathogenesis of DR also includes apoptosis of endothelial cells, and both factors lead to impairment the of blood–retinal barrier.

**Fig. 2:**
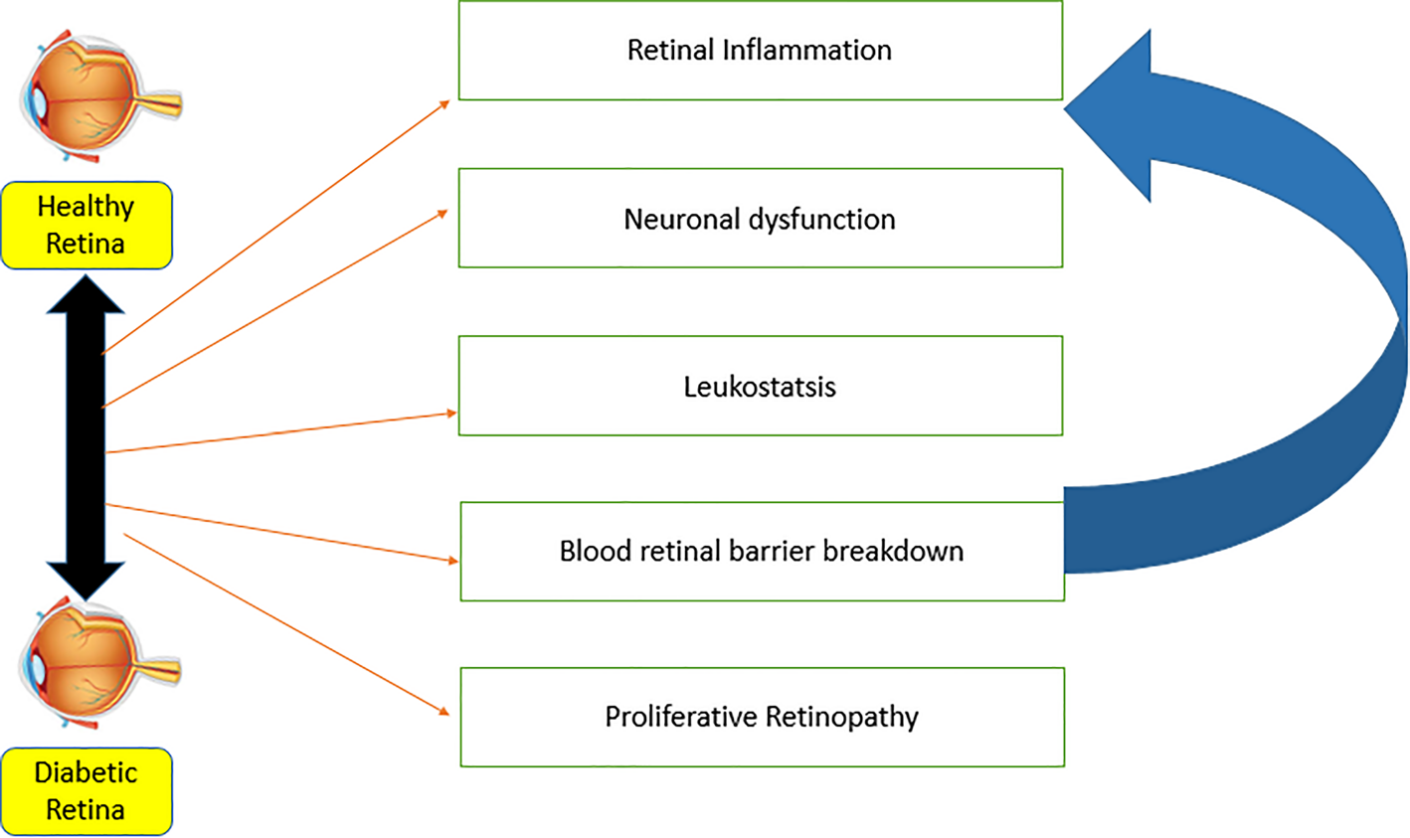
Molecular mechanism of diabetic retinal neuro degeneration

Some studies suggest that the increase in phospholipase A2 (PLA2) under diabetic conditions upregulates vascular endothelial growth factors (VEGF) [49], and that antigenic factors, including VEGF, trigger the changes in the microvasculature that cause DR [50]. Retinal neurodegeneration is a key element in the progression of DR. In a study on diabetic rats, apoptosis of retinal neurons was observed after 1 month of diabetes [51], and mitochondrial dysfunction and an increase in pro-apoptotic molecules have been observed in other diabetic animals [52]. In vitro studies of diabetic retinopathy have shown that increased glucose levels are associated with increased mitochondrial fragmentation and cell apoptosis. Oxidative stress is also associated with diabetic retinal degeneration, whereby reactive oxygen species are significantly lower in the retina of diabetic mice [53]. In addition, there is evidence that retinal neurodegeneration may occur independently of the pathophysiology of DR, as retinal thinning has been diagnosed in diabetic patients with little or no DR [54]. However, further work is needed to understand the phenomena of retinal neuro degeneration [55] (Fig. 2).

#### Inflammation in DR and the role of cells and cytokines in DR

Inflammation plays a central role in the pathogenesis of diabetic retinopathy and contributes to the development and progression of retinal damage in people with diabetes. Chronic, low-grade inflammation, which occurs in both the early and advanced stages of diabetic retinopathy, is associated with various inflammatory mediators, immune cells, and altered signaling pathways in the retina. The intricate interplay between inflammation and retinal dysfunction emphasizes the complexity of diabetic retinopathy and provides valuable insights for the development of targeted therapeutic approaches [56]. Chronic, low-grade inflammation is a characteristic feature of diabetes and is closely related to the pathological processes underlying diabetic retinopathy. Increased levels of proinflammatory cytokines such as tumor necrosis factor-alpha (TNF-α), interleukin-1 beta (IL-1β), and interleukin-6 (IL-6) have been detected in the vitreous and retina of people with diabetic retinopathy. In addition, increased expression of adhesion molecules and chemokines, including intercellular adhesion molecule-1 (ICAM-1) and monocyte chemoattractant protein-1 (MCP-1), contributes to the inflammatory milieu in the diabetic retina [57]. Infiltration of immune cells: the inflammatory environment in the diabetic retina promotes the infiltration of immune cells, including monocytes, macrophages, and leukocytes [58]. This influx of immune cells exacerbates retinal inflammation and contributes to vascular dysfunction, breakdown of the blood–retinal barrier, and the release of other pro-inflammatory messengers. Studies have shown the presence of activated microglia, the immune cells that reside in the retina, in areas of retinal damage, suggesting that they contribution to the inflammatory response in the diabetic retinopathy [59]. Inflammatory mediators in diabetic retinopathy lead to dysfunction in the endothelial cells of the retina, including increased expression of adhesion molecules and activation of pro-inflammatory signaling pathways [60]. This endothelial dysfunction contributes to microvascular abnormalities, such as capillary degeneration, increased vascular permeability and the formation of microaneurysms. The impaired integrity of the blood–retinal barrier leads to extravasation of plasma components, inflammation-induced oedema, and accumulation of exudates in the retina [61]. Microglia, the resident immune cells of the retina, play an important role in the inflammatory response associated with diabetic retinopathy [62]. Under diabetic conditions, microglia are activation and polarized towards a pro-inflammatory phenotype, releasing cytokines, chemokines, and reactive oxygen species. This increased microglial activation contributes to neuroinflammation, neuronal damage, and the amplification of the inflammatory cascade in the diabetic retina [63].

### Diagnosis of diabetic retinopathy

Inflammation is an important factor in the pathogenesis of DR, and many studies have found low-grade chronic inflammation in diabetic patients. Leukocytosis has also been found in the early stages of DR, [64]. In rats with diabetes, an increase in leukocytosis has been associated with damage to the blood–retinal barrier and endothelium. Chemokines attract and activate leukocytes, and elevated levels of chemokines such as macrophage inflammatory protein 1-alpha (MIP-1 *a)* and MIP-1*B* have been found in patients with diabetes [65]. It has been shown that MIP-1 deficiency leads to retinal leakage in diabetic mice. It has also been shown that inflammatory cytokines such as interleukin (IL-6), Il-1B, IL-8, and tumor necrosis factor (TNF-a) are significantly increased in diabetic patients [66].

Numerous methods for diagnosing DR can be found in the literature. The long-established basic diagnostic methods include visual acuity tests and dilated eye examinations. Visual acuity tests measure how well the eyes focus at near and far distances [67], and can be used to diagnose visual impairment and other eye abnormalities. During a comprehensive dilated eye examination, eye drops are used to prevent the pupil from contracting. This allows the doctor to examine the retina in detail under light. Both methods have the advantage that they are inexpensive and require little modern equipment. However, additional tests are usually required for a complete diagnosis.

In that context, fluorescein angiography enables the doctor to see the blood vessels at the back of the eye very clearly [68]. In a fluorescein angiogram, a fluorescent dye that is visible in blue light is injected into a vein in the arm. The dye circulates through the person’s bloodstream, including through the blood vessels of the retina. Shortly, after the dye is injected, a rapid sequence of photographs is taken of the retina, choroid, optic disc, iris, or a combination of these areas [69]. The dye in the blood vessels fluoresces and highlighting the vessels. Fluorescein angiography is particularly useful in the diagnosis of macular degeneration, blocked retinal vessels, and DR. This type of angiography is also used to assess people who may need laser surgery on the retina.

With Indocyanine green angiography, doctors can see the blood vessels in the retina and choroid [70]. As with fluorescein angiography, a fluorescent dye is injected into a vein, but the dye used enables a more detailed visualization of the blood vessels in the choroid than with fluorescein angiography. Indocyanine green angiography is used to determine macular degeneration and to detect the development of new blood vessels in the eye [70].

Optical coherence tomography is another examination in which images of the retina are taken, and in particular to measure the thickness of the retina [70]. This makes it easier to detect the presence of fluid in the retinal tissue and also provides information about leaks in the retinal tissue. OCT examinations can also be used at a later stage in DR to determine the effectiveness of the treatments carried out [71].

### Treatments for diabetic retinopathy

In the early stages of DR, i.e., mild or moderate, non-proliferative DR, doctors usually try to limit the progression of the disease by encouraging the patient to regulate their blood sugar levels [72]. However, more invasive treatments are indicated for advanced DR or macular oedema. Currently, laser photocoagulation is the main treatment used by ophthalmologists to control the development of neovascularization and macular oedema [73]. This treatment is widely accepted worldwide due to its significant short- and long-term effect of photocoagulation. Another treatment is the injection of endothelial growth factor inhibitors into the vitreous humor of the eye. These drugs stop the growth of regenerative blood vessels and thus reducing the accumulation of fluid [74]. Pan-retinal photocoagulation is another treatment that shrinks abnormal blood vessels and reduces eye infections [75]. Vasectomy, on the other hand, involves removing the blood from the center of the eye through a tiny incision. Advanced treatment methods today also include nanotechnology, stem cell technology, and advanced genomics and proteomics, which have been shown to significantly reduce the prevalence of DR [76] (Fig. 2).

## Nuclear protein

Nuclear proteins are proteins that are located inside the cell nucleus. Nuclear proteins are transported within the pore complex of the cell nucleus, which acts as a barrier between the nuclear membrane and the cytoplasm [77]. The export and import of proteins within the nuclear pore complex plays a very important role in many human biological functions and in the regulation of genes [78]. Nuclear proteins encode the specific amino acid sequences that function for protein based on protein localization. In particular, nuclear proteins are considered a class of binding proteins. Different types of nucleic acid (ribonucleic acid or nucleic acid) contain different types of protein, but [79]. Deoxyribose nucleoproteins, for example, are a combination of DNA and protein: complexes of genomic DNA wrapped around the group of histone proteins in the eukaryotic cell [80]. The most ubiquitous form of proteins in the cell nucleus is histones and protamines. These are found in chromosomes, and whole chromosomes are the houses of deoxyribo-nucleoproteins [81]. In addition, these proteins form complexes to form a multiprotein complex that interacts with DNA in the loop. These proteins also co-operate in the regulating of important cellular phenomena, such as DNA replication, DNA transcription and others. Another process regulated by these proteins is homologous recombination [82]. This process limits mutations and thus protects against life-threatening diseases. This phenomenon is also known as the DNA repair mechanism [83]. Different copies of recombined proteins interact with a standard DNA as part of the DNA repair mechanism. Therefore, deoxyribonucleic proteins are an important r player in the regulating of important events in the human cell life cycle [84].

**Fig. 3:**
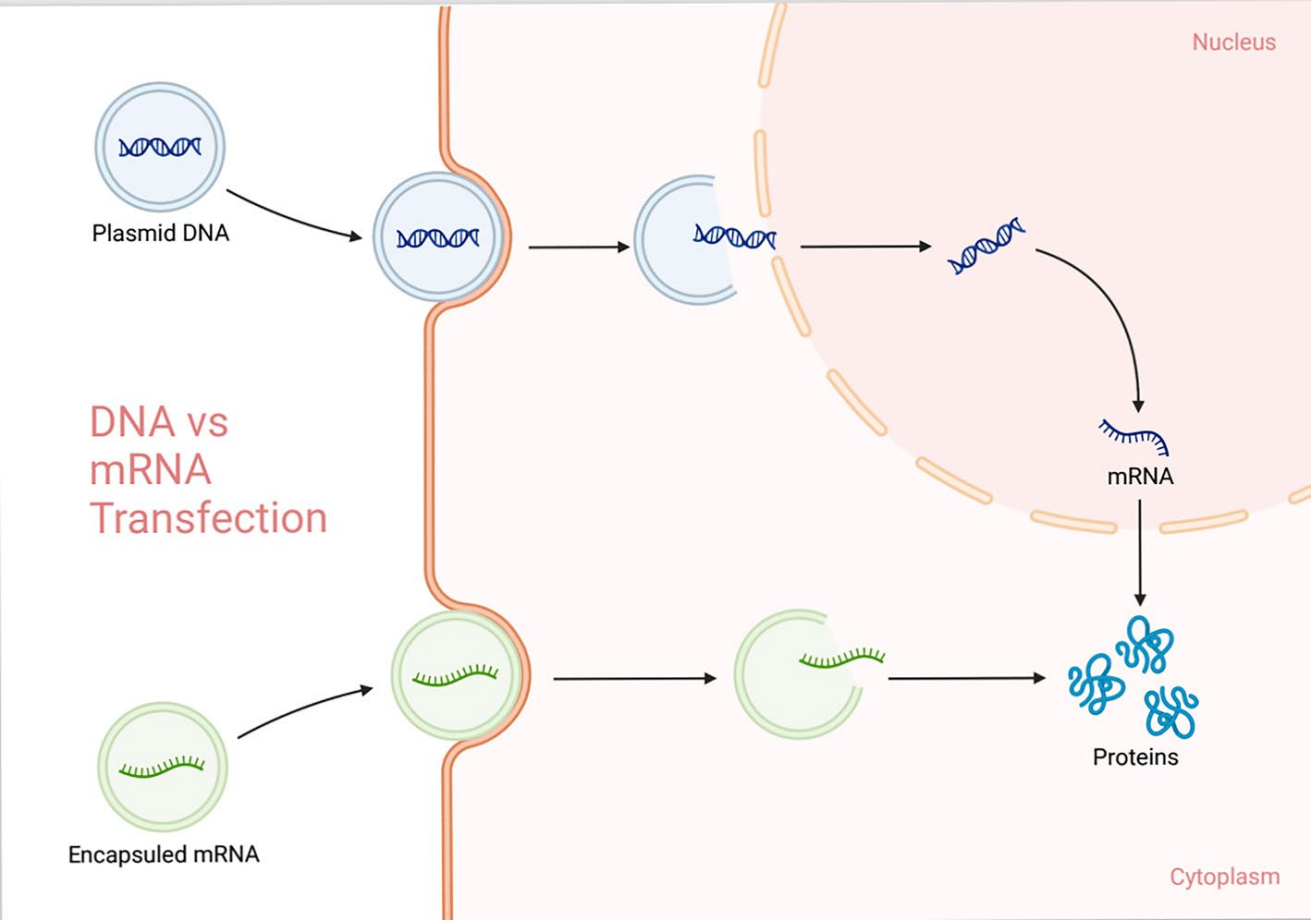
Nucleic acid protein synthesis process

Rib nucleoproteins are complex RNA-binding proteins and ribonucleic acid. These proteins are involved in several biological functions, including gene expression, transcription, translation, and the regulating of RNA metabolism [85]. The main function of the rib nucleoprotein is the RNA-binding motif. The Rib nucleoprotein is also known with rib nucleoprotein particles. These particles are involved in post-transcriptional regulatory processes, which mean that rib nucleoproteins are also crucial for important cell cycle functions. Most importantly, these proteins play a significant role in the translation process [86] (Fig. 3).

Histone is the most important nuclear protein and consists of basic amino acids, such as lysine or arginine [87]. Another basic nuclear protein is protamine, which consists of aspartic acid and glutamic acid. While histone is positively charged, protamine is negatively charged [88]. Histone is important for the structural support of chromosomes and regulates genetic expression. Eight histone proteins are linked together to form a nucleosome. Histones are important for the organized genome and the packaged genome in a human cell. Histone proteins are also responsible for gene expression and indicate whether a gene is switched on or off [89]. Histone proteins are, therefore, a hallmark of gene expression. Protamines, on the other hand, are small nuclear proteins, such as arginine, which replace histones during spermatogenesis. Protamines enable the packaging of DNA, but in the genetic data, this protein is decompressed for protein synthesis [90].

### Nuclear protein in diabetic retinopathy

Several nuclear proteins have been reported to play a role in the pathogenesis of DR and are, therefore, of interest when considering new treatments for the disease. For example, increased expression of S protein has been reported in DR [91], and Protein Kinase R (PKR) has also been reported in the early stages of DR [92]; this has prompted scientists to investigate the cellular localization of PKR. In one such study, diabetic and normal rats were injected with streptozotocin (STZ), and then, their retinas were examined for the cellular localization of PKR 3, 6, and 35 days after injection. PKR was analyzed in the nuclei of INL and RGCs [93]. In this study, although no change in the cellular localization of PKR was observed in the retinas of normal or diabetic rats, a higher nuclear expression of PKR was observed in the retinas of diabetic rats on day 3 than on day 6, and the lowest expression was observed on day 15 [94]. Similar results were found for another protein, the activated eIF2-α protein (p-eIF2-α): on day 3, this protein was highly expressed, but the expression decreased significantly by day 35 [95]. RNA-dependent proteinase kinase (PKR) is a serine–protein kinase that develops a stress response and regulates pro-inflammatory signaling pathways, such as retinal ganglion cells and apoptosis, particularly in patients with Huntington’s disease and Parkinson’s disease [96]. PKR is important in cells due to its role in antiviral defense. The activation of PKR in response to stress leads to cell suppression, cell proliferation, and apoptosis [97]. The PKR-linked protein (RAX) is known as a direct activator of PKR and serves to regulate apoptosis by activating PKR under stress conditions [98]. In another study, expression levels, subcellular localization, and PKR activation were observed in the retina of normal and diabetic rats. According to this study, hyperglycemia-induced stress mediates RAX expression and deregulates RAX function [99].

Protein S (PS) is a 75-kDA vitamin K-dependent glycoprotein that modulates inflammation by inhibiting the coagulation system and the expression of inflammatory cytokines. PS modulates apoptosis and inflammation by binding to the tyrosine kinase Mar TAM, Tyro3, and other receptors [100]. In transgenic mice, it was found that the freely circulating PS level stops the apoptosis of pancreatic B cells and improves the pathogenesis [101]. In addition, administration of the PS protein in diabetic mice leads to renal dysfunction. Vascular events, including pulmonary embolism and deep vein thrombosis, were found more frequently in patients suffering from PS deficiency. Another study emphasizes the anticoagulant function and clinical relevancy of PS [102]. PS also has a protective effect on the eyes of patients suffering from DR.

### Mechanisms of action of nuclear proteins in diabetic retinopathy

Nuclear proteins are essential for the development of diabetic retinopathy, a common and potentially blinding disease. In the cell nuclei of the retina, these proteins regulate gene expression and modulate cellular responses. Diabetic retinopathy is associated with deregulation of many nuclear proteins that cause molecular and cellular changes. The transcription factor NF-κB regulates genes involved in inflammation, immunological responses, and cell viability. In diabetic retinopathy, NF-κB activation in retinal cells is associated with persistent, low-grade inflammation. Increased NF-κB activity can lead to upregulation of pro-inflammatory genes, which in turn induce cytokines, chemokines, adhesion molecules, and other mediators that cause microvascular dysfunction, breakdown of the blood–retinal barrier and progression of retinal injury [103]. The enzymes known as HDACs in the cell nucleus control the acetylation of histone proteins and thus influence chromatin structure and gene expression. Diabetes-related changes in gene expression in retinal cells due to dysregulation of HDAC activity have been associated with vascular dysfunction, oxidative stress, and angiogenesis. HDACs modulate histone acetylation, an epigenetic process that may affect diabetes-related retinal disease genes [104]. The molecular and cellular changes in diabetic retinopathy are mediated by these key proteins and signaling pathways. Their dysfunction leads chronic inflammation, oxidative stress, vascular dysfunction, and pathological angiogenesis in the retina, leading to microaneurysms, hemorrhages, exudates, and neovascularization [105].

### Roles of miRNAs and long noncoding RNAs in the progression of diabetic retinopathy

MiRNAs play a very important role the regulating of various processes in retinal cells, including apoptosis, migration, proliferation, and others [106]. The study reported on the role of miRNAs in the regulating DR associated with NV. In the early stages of DR in patients with type 2 diabetes, the study found that elevated glucose levels have severe effects on retinal pigment epithelial cells, human retinal endothelial cells (ECs), and key components of the blood–retinal barrier [107]. In other studies, retinal pigment epithelial cells and human retinal ECs were also analyzed to determine the role of miRNA in DR [108], and retinas from STZ-induced diabetic rats were used to try to determine the set of miRNAs that serve to alter the expression of retinal ECs. MiRNA profiling also revealed that compared to control rats, 80 miRNAs showed increased expression in diabetic rats, while six showed reduced expression [108, 109]. These miRNA data, therefore, provide an initial overview of the signaling pathways that lead to increased pathogenesis of DR after 3 months of diabetes. Another study investigated the expression of lncRNA by profiling diabetic mice treated with STZ injection [110]. In this study, 303 IncRNAs were found to be differently expressed in the retinas of rats with diabetic retinopathy: 214 were downregulated and 89 were upregulated [111]. Based on this, the study carried out a pathway analysis and found that the mRNAs expressed by the IncRNAs include the MAPK signaling pathway, axon guidance, chemokine signaling, pyruvate metabolism, and coagulation cascades. All of these signaling pathways are associated with the pathogenesis of DR, neurodegeneration, inflammation, and other diseases [111, 112].

### The role of other nuclear proteins

STAT proteins are elevated in DR and also in DR-related mechanisms at early stages of the disease [113]. These proteins with the unique, comprehensive and powerful induce the disease progression. Researchers have identified a link between the STAT proteins, microRNA, and IncRNAs. Numerous factors, including miR-19b, miR-132, miR-05, and others, are known to promote the STAT protein activity. In the study, an increased level of STAT protein was found in DR, while miRNAs decreased significantly [114].

### The potential of nuclear proteins in the treatment of diabetic retinopathy

#### Mitogen-activated protein kinase (MAPK)

In chronic diabetes, protein kinase (PKC) modulates the vascular function by interacting with several other signaling complexes in vascular ECs. The MAPK pathway plays an important role in the complications associated with diabetes and the activity of this pathway completely depends on the activation of PKC [115]. The MAPK family includes extracellular signal-regulated kinase (EPK), and many stress-activated components. The study reported that activation of MAPK synthesis glucose-induced EMC protein. Some other proteins, such as transcription factors NF-κB, are also activated by MAPK phosphorylation [116]. High glucose level was normalized by e inhibiting either PKC or MAPK. Inhibition of PKC cells due to high glucose levels causes a reduction in MAPK activation. The expression of EPC proteins in kidney cells was also observed with MAPK activation. However, MAPK activation occurs via the PKC-independent pathway [117].

#### Transcription factors

**Fig. 4:**
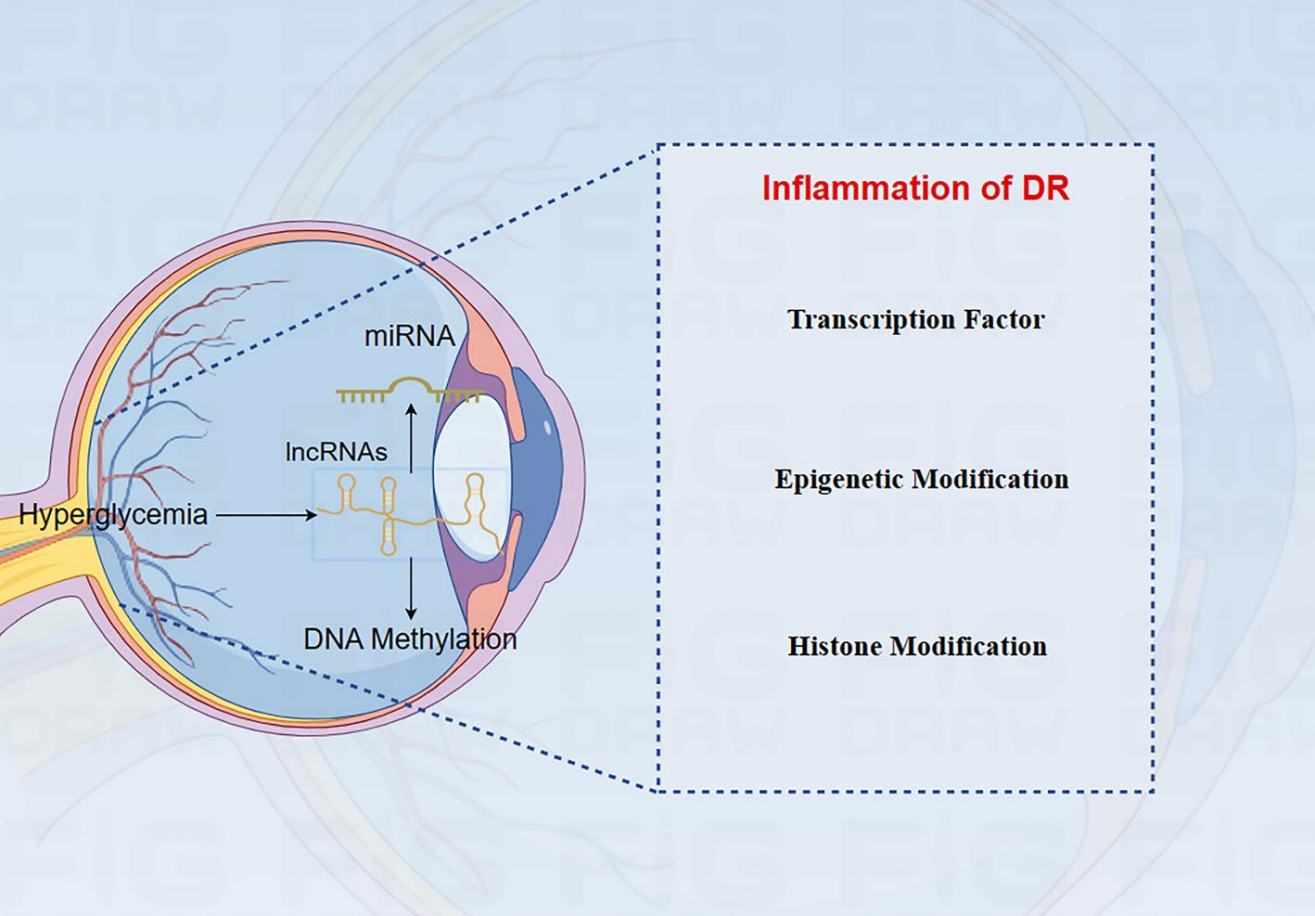
Various proteins' roles in the development of diabetic Retinopathy

Extracellular and intracellular signals contribute to transcription factors modulating numerous aspects of diabetes. Two transcription factors in particular, AP-1 and NF-Kb, mediate the effect of diabetes. In unstimulated cells, these proteins act as a dimer in the cytoplasm, but upon stimulation, IkB kinase (IKK) is activated and mediates phosphorylation leading to degradation of IkB. The study reported that oxidative stress is the main activator of NF-KB. In addition, glucose-based ET-1 expression regulates the NF-kB activation [118]. Transcription factor-based proteins that are activated during hyperglycemia include Jun, FOS, and ATF, which contribute to the synthetization of AP1 dimers, activate multiple stimuli, and regulate various functions. The MAPK-mediated EMC protein in ocular cells is dependent on both AP-1 and NF-kB [119]. By activating these protein transcription factors, ET-1 increased FN expression. This increased expression was observed in the retina, kidney, diabetic complications, and heart. Increased oxidative stress activates NF-kB, which is also a redox-sensitive transcription factor. Numerous transcription factors were important for these signaling pathways [120] (Fig. 4).

#### Transcription co-activators

The histone protein is packaged with the genomic DNA and plays a very important role in gene regulation. The remodeling of chromatin in the cell nucleus is regulated by the deacetylation and acetylation of histone residue [121]. It is crucial for enabling access to the transcription factor during DNA binding. After nuclear translocation, transcription factors, such as NF-kB remain inactive. P300 regulates NF-kB activity in diabetes [122]. The expression of FN was detected in retinal cultures and in diabetic rats. Histone deacetylation regulates several signaling pathways [123], but the signaling pathway can be modified with protein kinase B, protein kinase C, and other proteins. Meanwhile, the results of another study showed that molecular changes caused by diabetes induce activation of PKC and MAPK, which then leads to increased expression of ECM protein [124].

## Conclusion

Diabetic retinopathy, which is associated with diabetes, leads to blindness and vision loss. The most important risk factors include poor blood sugar control, the duration of diabetes, and high blood pressure. Worldwide, about 27% of diabetics have diabetic retinopathy, but regional prevalence varies widely. For example, while the prevalence in Africa’s is 31.6%, in Ethiopia, it is 19.48%. Diagnosis includes comprehensive eye examinations with ophthalmoscopy, fluorescein angiograms, and optical coherence tomography microvascular. It appears that nuclear proteins have a significant impact on DR development. Nuclear proteins are integral components of the nuclear pore complex, which serves as a physical barrier between the nuclear membrane and the cytoplasm. Several nuclear proteins have been linked to the development of diseases, including DR. PKR, a nuclear protein kinase, plays a crucial role in the initial stages of DR. MicroRNA (miRNA) plays a crucial role in regulating various processes in retinal cells, such as apoptosis, migration, and proliferation. Elevated levels of STAT proteins were observed in both early and later stages of DR. Taken together, these proteins appear to have a clear, broad and strong impact on the progression of DR. Therefore, they have great potential as a target for the development of novel therapeutic interventions for this condition.

## Data Availability

Not applicable.

## Abbreviations

NPDR: Non-proliferative diabetic retinopathy
PDR: Proliferative diabetic retinopathy
VEGFC: Vascular endothelial growth factor C
VTDR: Vision-threatening Diabetic Retina
PLA2: Phospholipase A2s
MIP-1 a: Macrophage Inflammatory Protein 1-Alpha and MIP-1B
TNF-a: Tumor necrosis factor
BRB: Blood–Retinal Barrier
STZ: Streptozotocin
MAPK: Mitogen-Activated Protein Kinase
PKR: Protein Kinase R
