# Correction: Nuclear proteins and diabetic retinopathy: a review

**DOI:** 10.1186/s12938-024-01293-1

**Published:** 2024-10-10

**Authors:** Bin Li, Wahab Hussain, Zhi-Liang Jiang, Jia-Yi Wang, Sarfraz Hussain, Talat Bilal Yasoob, Yuan-Kun Zhai, Xin-Ying Ji, Ya-Long Dang

**Affiliations:** 1 Department of Ophthalmology, The First Affiliated Hospital, Henan University, Kaifeng, 475004 Henan China; 2 School of Stomatology, Henan University, Kaifeng, 475000 China; 3 Kaifeng Municipal Key Laboratory for Infection and Biosafety, Henan International Joint Laboratory of Nuclear Protein Regulation, School of Basic Medicine Science, Henan University, Kaifeng, 475000 China; 4 School of Clinical Medicine, Henan University, Kaifeng, 475004 Henan China; 5 San-Quan College, XinXiang Medical University, No. 688 Xiangyang Road, Hongmen Town, Hongqi District, Xinxiang City, 453003 Henan China; 6 College of Environment, Hohai University, Nanjing, 210098 China; 7 Department of Animal Sciences, Ghazi University, Dera Ghazi Khan, 32200 Pakistan; 8 Kaifeng Key Laboratory of Periodontal Tissue Engineering, Kaifeng, 475000 China; 9 Faculty of Basic Medical Subjects, Shu-Qing Medical College of Zhengzhou, Mazhai, Erqi District, Zhengzhou, 450064 Henan China; 10 Department of Ophthalmology, Sanmenxia Central Hospital, Henan University of Science and Technology, Sanmenxia, Henan China; 11 Department of Ophthalmology, Sanmenxia Eye Hospital, Sanmenxia, Henan China; 12 Department of Ophthalmology, Henan University of Science and Technology School of Medicine, Luoyang, Henan China

**Correction: BioMedical Engineering OnLine (2024) 23:62** 10.1186/s12938-024-01258-4

Following publication of the original article [1], the statement in the Funding information section was missing and should have been updated us below:

Funding

This work was supported by grants from the National Natural Science Foundation of China grant, China (no. 31902287 and 81670988), Foundation of Science & Technology Department of Henan Province, China (no. 212102310103), Natural Science Foundation of Education Department of Henan Province, China (no. 21A320004), Foundation of the National Health Commission of Henan Province, China (no. Wjlx20), Foundation of the Educational Administration Department of Henan University, China (no. HD20017, XJJG2020-83), Medical Science and Technology Development of Henan Province, China (no. LHGJ20210569), Foundation of Science & Technology Department of Kaifeng City, Henan Province, China (no. 2203015 and 2203035).

The original article has been corrected.

## References

[1] Li B, Hussain W, Jiang ZL, Wang JY, Hussain S, Yasoob TB, Zhai YK, Ji XY, Dang YL. Nuclear proteins and diabetic retinopathy: a review. BioMed Eng OnLine. 2024;23:62. 10.1186/s12938-024-01258-4. 38918766 10.1186/s12938-024-01258-4 PMC11197269

